# Interventions against hearing loss as an integral component of successful tinnitus therapy

**DOI:** 10.1007/s00106-023-01331-9

**Published:** 2023-10-04

**Authors:** Gerhard Hesse, Georg Kastellis

**Affiliations:** 1 Ohr- und Hörinstitut und Tinnitus-Klinik, Krankenhaus Bad Arolsen, Große Allee 50, 34454 Bad Arolsen, Germany; 2 Universität Witten-Herdecke, Witten, Germany

**Keywords:** Otitis media, Hearing aids, Cochlear implants, Sensation disorders, Hearing disorders

## Abstract

Tinnitus very often develops from acute or chronic hearing loss, mainly inner ear deafness. The frequency of the tinnitus mostly corresponds to the frequency range of the hearing loss and is enhanced by down-regulation of inhibition in the central auditory pathway for these frequencies, in addition to focused attention and enhanced arousal for the disturbing sound. Therefore, interventions to improve hearing such as mid-ear surgery or—more often—electronic devices including hearing aids or cochlear implants (CI) are important for the treatment of tinnitus. In this review, the current German S3 guideline “Chronic tinnitus” and recent literature are discussed.

Ringing in the ears or tinnitus very often occurs in the wake of or as a concomitant symptom of hearing loss, regardless of whether this occurs acutely or is chronic and progressive. Moreover, the frequency or frequency band of tinnitus lies almost always in the range of the greatest hearing loss [5, 8]. Thus, in a sense, tinnitus corresponds to a phantom noise in the auditory cortex: In this context, a major cause of the perception of ringing in the ears is a central downregulation of inhibition for these frequencies, accompanied by cortical reinforcement mechanisms, which in turn are accentuated by increased focusing and attention responses [15, 19, 21].

Compensating the actual hearing loss can therefore often reduce tinnitus perception and reduce distress [16]. If the hearing loss is middle ear related (otosclerosis, chronic otitis media), surgical interventions are certainly the best choice. However, for the much more common forms of tinnitus caused by permanent damage to the hair cells of the inner ear, curative therapy is not possible, neither surgically nor with medication [7]. Compensation of the hearing loss is therefore only possible by means of modern hearing aids or cochlear implants. For the reduction of tinnitus perception and distress by hearing-improving interventions, studies have been presented for many years, albeit with moderate evidence [17, 20, 25].

## Importance of hearing loss for tinnitus distres

The guideline “Chronic Tinnitus” [4], updated in September 2021, states: “Hearing aids should be recommended for chronic tinnitus and hearing loss.”

According to the guideline, there are only papers with moderate or weak strength of evidence for the effectiveness of hearing aids in tinnitus therapy, which is largely due to the fact that only a few studies evaluate the effectiveness of hearing aids alone for the treatment of chronic tinnitus. Nevertheless, a great many studies confirm that hearing aids promote tinnitus suppression and habituation.

The effects of any therapy on tinnitus burden are measured in international studies with the Tinnitus Handicap Inventory (THI) or the Tinnitus Functional Index (TFI). The THI measures perceived tinnitus burden in 25 questions and is particularly well suited for assessing psychotherapeutic approaches, whereas the TFI can be used to calculate an index from at least 19 questions to identify the severity of tinnitus burden in different areas of life.

Hearing aids promote tinnitus suppression and habituation

For example, a recent Swedish study examined the effect of hearing aids on a total of 100 patients, 50 of whom had hearing loss with tinnitus and 50 of whom had hearing loss without tinnitus. Of these 100 participants, data from 92 could eventually be analyzed, 46 from each group. For the patients with tinnitus and hearing loss, significant improvement in THI was achieved with hearing aid application.

Both groups, including the non-tinnitus patients, also improved in tests that assessed cognitive function (reading span test and hearing-in-noise test; [29]).

**Fig. 1:**
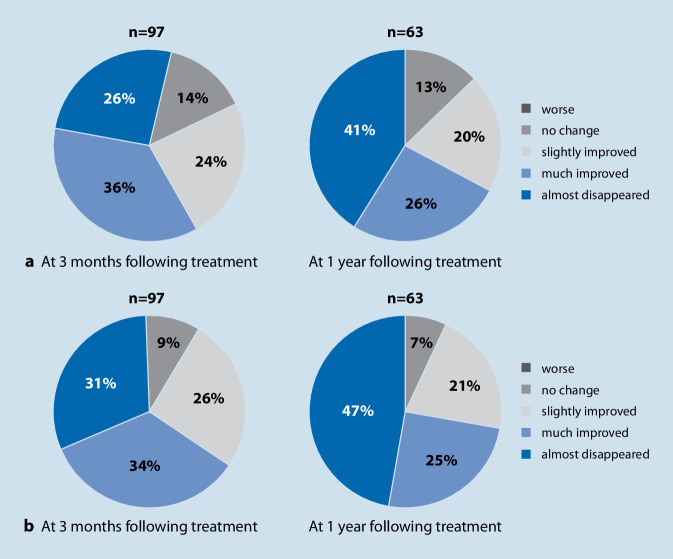
Unilateral hearing aid fitting for hearing loss and tinnitus, 3 months and 1 year later (improvement measured with the Tinnitus Handicap Inventory). Modified after [26]

A study from Japan also investigated comparable effects for unilateral hearing loss with unilateral tinnitus alone. For this purpose, 97 patients were fitted with hearing aids on the hearing-impaired and tinnitus-affected side and questionnaires were administered at baseline, after 3 months, and after 1 year. The values in the THI improved significantly as early as after 3 months and then again after 1 year; the tinnitus loudness was also significantly reduced by wearing the hearing aid and, overall, 80–90% of the patients improved significantly. None of the patients worsened because of hearing aid fitting (Fig. 1; [26]). This study highlights the great influence of hearing loss regarding the actual burden of tinnitus.

## Hearing aids also for mild hearing loss

Fitting of hearing aids is generally carried out in accordance with the applicable national guidelines for hearing aids. In individual cases, however, especially in the case of isolated high-frequency hearing loss and high-frequency tinnitus, a hearing aid fitting can be useful even without the presence of a hearing loss corresponding to these guidelines.

The experience of hearing aid fitting is very positive even for mild hearing loss, especially in the high-frequency range, because modern technology enables good amplification of high frequencies, which leads to an improvement in hearing in the tinnitus frequency and is therefore also very well accepted by those affected.

Early hearing aid fitting is of increasing importance even for mild and moderate hearing loss

For tinnitus treatment, early hearing aid fitting, even in the case of mild or moderate hearing loss, is also of increasing importance, as recent studies have shown: In a Korean study, 114 patients with tinnitus were randomized into three groups and fitted with different types of hearing aids. As no further counseling or education took place, a pure hearing aid effect could be demonstrated, leading to a significant improvement in the THI burden after 3 and 6 months [28]. Especially the sole therapeutic use of hearing aids in these patients increases the evidence of the study, even though no “placebo group” was included.

A Danish study also investigated the effect on tinnitus of amplification by means of hearing aids in patients with high-frequency hearing loss. For this purpose, 23  patients with tinnitus and high-frequency hearing loss (> 3 kHz) were included. They were randomized to receive two different hearing aid treatments for 3 months. One group was set with broadband amplification in the frequency range from 125 Hz to 10 kHz, while the other group had amplification only in the low-frequency range (< 3–4 kHz). Evaluation was performed with the THI and the TFI, and loudness was recorded with visual analog scales. In fact, there was a significant difference between these two groups, with the broadband amplification, especially in the high-frequency range, being clearly superior to the low-frequency amplification in terms of tinnitus distress as well as in terms of tinnitus loudness. The tinnitus frequency did not change with the treatment [13].

**Fig. 2:**
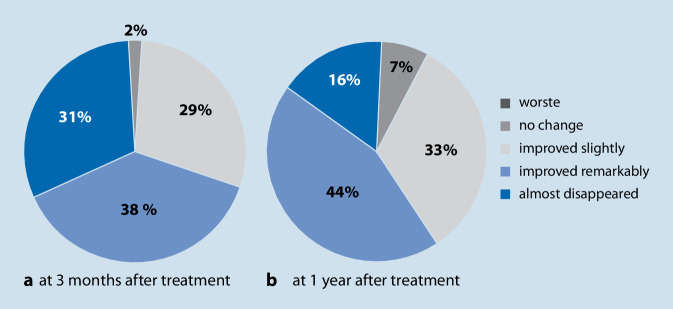
Changes in tinnitus exposure in patients with mild high-frequency hearing loss 3 months and 1 year after treatment with hearing aids alone. Modified according to [24]

In a Japanese study of 91 patients, the effect of hearing aid fittings was recorded in patients with low-grade, high-frequency-related hearing loss of less than 30 dB who had no subjective hearing impairment. The corresponding questionnaires were evaluated. Initially, 103 patients (72 men, 31 women) were recruited, ten returned the hearing aids, five because their ear noise had become significantly less distressing and five because they could not tolerate the hearing amplification and were mainly bothered by the intrinsic noise of the hearing aid. Another two participants were excluded because the questionnaires had not been properly completed. All scores in the THI decreased significantly for the remaining 91 patients, and the visual analog scales for tinnitus distress also decreased significantly (Fig. 2). Overall, 90% of patients felt a significant improvement in tinnitus distress [24]. The study shows that tinnitus distress is reduced even when there is only a minor hearing loss, usually high-pitched, where subjectively the hearing loss is not even perceptible to the patients.

## Importance of quality of hearing aid fitting

Unfortunately, most studies investigating the effect of hearing aids on tinnitus perception and distress are methodologically at least heterogeneous, and in most cases, tinnitus is not the main complaint and thus not the starting point for hearing aid fitting. This is the result of a recent review from Greece [14], which analyzed 34 studies in more detail. However, all studies found that hearing aid fitting has a positive effect on tinnitus perception, especially in patients suffering from hearing loss. However, no robust conclusions could be drawn from the studies. The authors conclude that in future studies the basic conditions of the therapeutic approach must be recorded more precisely and, above all, the final measurement points must be defined more accurately. Only then can high-quality evidence be obtained on whether hearing aids should be used in tinnitus treatment [14].

Quality of fitting and hearing gain are essential for reducing tinnitus burden

A meta-analysis from Sweden evaluated 27 studies in which tinnitus patients were examined before and after hearing aid fitting. A total of 1400 participants were included in this meta-analysis. While there was a highly significant improvement in the loudness of tinnitus, the reduction in tinnitus distress was significant, although not as much. However, when only the studies in which hearing aid amplification actually improved hearing were evaluated, this also resulted in a significantly greater reduction in tinnitus stress. The authors conclude that especially a successful hearing aid fitting, which also brings an improvement in terms of hearing loss, can effectively reduce tinnitus stress [27]. This means that the quality of the fitting and thus the actual hearing gain are also essential for a reduction in tinnitus distress.

The quality of the fitting is essential for a reduction in tinnitus distress

Overall, however, there is a lack of convincing studies and meta-analyses supporting the evidence of the efficacy of hearing aids alone, for the systematic reasons outlined above. Accordingly, Hoare and coworkers [11] concluded in their Cochrane Review that a recommendation on the use of hearing aids for the indication of tinnitus could not be made because of the poor methodology of the studies. In a 2018 update of this Cochrane Review [22], this assessment is maintained, but overall, after evaluating studies that compared noisers (noise generators) and hearing aids, an efficacy for hearing aids is generally confirmed.

## Effect of hearing aids on cognitive abilities

As proven by numerous longitudinal studies, successful and early provision of hearing aids also has positive effects on cognitive abilities and slowdown of the development of dementia. Cited here is only one recent study from Greifswald, which followed up 258 patients (older than 70 years) over 2 years regarding the development of cognitive deficits. Overall, 123 patients were hearing impaired, of whom 54 (43.9%) wore hearing aids. The symptomatology of those who wore hearing aids worsened significantly less, even though preexisting cognitive deficits did not change. Quality of life also improved in the hearing aid users compared to the others, but only in the first year [2].

## Noiser/noise generators

Sound carriers with noise or a noise generator (“noiser”) are also discussed for tinnitus suppression, often in combination with a hearing aid.

The aforementioned Cochrane analysis from 2018 [22] evaluated eight studies with a total of 590 participants regarding the efficacy of noise therapies, mediated either by hearing aids or by sound generators. The criticism is that there was virtually no blinding and a high risk of bias in all studies. In particular, the comparison of the use of hearing aids and noisers showed no significant effects with respect to noiser use. Evidence of measurable superiority of sound therapy or noiser treatment over placebo or education and counseling was not found for any device tested. Overall, the quality of the studies was weak, especially because no second or subsequent effects were recorded regarding depression or anxiety. The general quality of life was also not considered in the studies. The authors concluded that this Cochrane analysis does not provide any evidence that noise or sound therapy for tinnitus is superior to general counseling or placebo treatment.

Furthermore, long-term effects of noiser treatment and thus possible damage to the auditory pathway by constant sound stimulation have not been investigated so far and thus have not been recorded, although they are possible [3, 18, 19, 21, 23].

The S3 tinnitus guideline does not recommend noise generators or noisers for chronic tinnitus

The S3 Tinnitus Guideline [4] therefore does not recommend noise generators or noisers for chronic tinnitus.

## Cochlear implants and tinnitus

For the provision of cochlear implants to the profoundly deaf, the effect on tinnitus is much better documented. A review from The Netherlands selected seven prospective cohort studies from a total of 4091 publications with a total of 105 patients aged 10–26 years. All tinnitus patients in this study had either asymmetric hearing loss or unilateral complete deafness. In all studies, there was a statistically significant improvement in tinnitus burden after cochlear implantation. The authors concluded that tinnitus, as a major complaint, can be positively improved by a cochlear implant (in the presence of relevant hearing loss; [1]).

A meta-analysis from Brazil screened 140 articles, and used 11 for meta-analysis. Six studies showed a high level of evidence for the effect of the cochlear implant on tinnitus distress, and another five studies found moderate evidence. In the studies with high-level evidence, a total of 136 patients were interviewed with the THI, and they achieved a statistically significant improvement in the postoperative THI score. The authors concluded that tinnitus patients can benefit from both unilateral and bilateral cochlear implantation, also regarding tinnitus distress [6].

In all cases, a positive effect on a preoperatively existing tinnitus is shown after provision with a cochlear implant. Conversely, however, in rare cases a new occurrence of tinnitus after implantation is also possible, whereby it cannot be determined with certainty whether the effect is due to the implantation itself or occurs spontaneously. The positive effect of a cochlear implant on tinnitus seems to be independent of age, i.e., even patients over 80 years of age may benefit from cochlear implantation for tinnitus and hearing loss.

According to the guideline [4], *cochlear implants should be recommended* for profoundly deaf and hard-of-hearing patients, including unilaterally deafened patients with tinnitus.

## Audiotherapy

Hearing therapy or audiotherapy can be used as a supportive measure. For this purpose, targeted exercises are used to train central auditory processing skills such as directional hearing, focusing, and differentiation in noise with and without hearing aids or a cochlear implant, and specifically overhearing of the tinnitus [9]. Hearing therapy can also improve the acceptance of hearing aids and thus promote the tinnitus situation. It can be conducted as manualized treatment; a meta-analysis presented in 2010 found weak evidence that hearing therapy interventions are effective [10].

## Discussion

Interventions to improve hearing ability are an essential element of tinnitus treatment to enable a central inhibition of the tinnitus frequency. In addition to surgical rehabilitation of the middle ear and, if indicated, cochlear implantation, the focus of hearing improvement is on adequate hearing aid provision. The quality of studies on the use of hearing aids in the treatment of tinnitus is improving, and several studies, including those presented here, show significant improvements in tinnitus distress for most patients. Very important in this context is the realization that the actual hearing loss must be sufficiently amplified [27]. This shows the close association of hearing loss with tinnitus distress. Depending on the study, 70% to almost 90% of patients benefit from a good hearing aid fitting. It is particularly difficult to provide hearing aids for patients with severe hearing loss, while patients with mild hearing loss benefit significantly regarding tinnitus burden, at least if they accept hearing aids.

Overall, most studies are methodologically very heterogeneous, especially regarding the parameters recorded and the end time measurement points, which clearly diminishes their evidence. Often, the studies also compare only certain types of hearing aids or hearing aids and sound generators, which then again does not allow any clear statements to be made as to whether hearing aids have a clear effect on tinnitus or not. Unfortunately, there is a large gap between clinical relevance, clinical effects, and the scientifically verifiable evidence.

Many patients are considered as “fitted” even if the hearing loss is not compensated effectively at all

However, a very important weak point of all studies in this regard is that the actual quality of hearing aid fitting is not recorded. Thus, many patients are considered to be “fitted” even if the existing hearing loss is actually not compensated effectively at all. In this case, the hearing aids are not worn; the reasons are often fittings that are too open or too little amplification in the affected frequency range. A positive effect on tinnitus perception then fails to materialize [12].

While in recent years many studies still used combination devices (hearing aid plus noiser), there is now an increasing number of studies for the effect of hearing aids alone, since, as the current guideline also confirms, there is generally no evidence for the use of combinations of hearing aids with noisers.

The effect of cochlear implants has been studied several times in recent years, most recently in detail in unilateral fitting and unilateral deafness or profound hearing loss. Even if new tinnitus may occasionally occur as a result of the surgery itself, the general effect is very positive with good evidence; furthermore, it effectively reduces the tinnitus burden because the auditory cortex is stimulated again and, as with a well-fitted hearing aid, central inhibition mechanisms can take effect again.

## Outlook

The low evidence of previous studies is mainly based on the fact that a hearing aid fitting is usually part of a (multimodal) tinnitus therapy, in which the audiological diagnostics and the subsequent counseling are already therapeutically effective. Furthermore, placebo hearing aid fitting is hardly feasible. Better evidence could only be obtained if hearing aid fitting was used as a stand-alone therapeutic measure and compared with a non-treated group with similar hearing loss. Primary and secondary treatment effects would need to be clearly identified and then measured prior to the studies. For the very common high-frequency hearing losses with high-frequency tinnitus, this would be quite possible, as shown in the study from Korea  [28]. For these patients, “placebo matching” would also be possible by deliberately down-regulating the amplification effect for these high frequencies in the comparison group. Patients with unilateral hearing loss could also possibly be compared as well fitted and unfitted, as shown in the study from Japan [26]. In the case of bilateral (centralized) tinnitus, therapy effects would be measurable by unilateral fitting compared to unfitted tinnitus patients, even if other therapeutic interventions were added.

## Practical conclusion

- Hearing loss leads to cortical amplification mechanisms for the missing frequencies and thus often to tinnitus amplification.
- Interventions to improve hearing, essentially adequate hearing aid fitting, compensate for hearing loss at the central level, thus requiring less cortical amplification effects in the auditory pathway. This in turn promotes inhibition and can thus also better suppress the tinnitus frequency.
- Hearing aids should therefore be prescribed early in cases of tinnitus, even in cases of high-frequency hearing loss.
- Hearing therapy measures can support the improvement of hearing ability and thus also the habituation of the ringing in the ears.

## Declarations

For this article no studies with human participants or animals were performed by any of the authors. All studies mentioned were in accordance with the ethical standards indicated in each case.

The supplement containing this article is not sponsored by industry.
